# Comparison of Endoscopic Versus Microscopic Tympanoplasty

**DOI:** 10.22038/IJORL.2022.61234.3104

**Published:** 2022-05

**Authors:** Iqra Zakir, Ahmad Nawaz Ahmad, Hamdan Ahmed Pasha, Shakil Aqil, Saeed Akhtar

**Affiliations:** 1 *Department of Otolaryngology – Head and Neck Surgery, Liaquat National Postgraduate Medical Centre, Karachi, Pakistan.*

**Keywords:** Tymapanoplasty with endoscope, Graft uptake, Hearing improvement

## Abstract

**Introduction:**

Tympanoplasty is a common surgery for chronic otitis media and has conventionally been performed with a microscope for decades. The trend of endoscopic minimally invasive surgeries has been increasing worldwide for the last few decades. Few studies have discussed the outcomes of tympanoplasty with microscope and tympanoplasty with endoscope . This study aims to compare results of tympanoplasty done with microscope vs endoscope in terms of graft take rate and improvement in conductive hearing loss.

**Materials and Methods:**

We did a retrospective review of 120 patients (54 male and 66 female) who underwent Type I tympanoplasty at Liaquat National Postgraduate Medical Center from January 2019 to January 2020. We included 60 patients who underwent tympanoplasty with microscope and 60 patients who underwent tympanoplasty with endoscope. Postoperative graft uptake and hearing improvement were studied.

**Results:**

Overall mean preoperative hearing loss was 30.24 (±9.61) dB as compared to mean postoperative hearing loss, which was reduced to 19.36 ( ±8.54) dB, and the difference was significant (P-value <0.001. No statistically significant difference was found for air-bone gap closure between the two groups (P-value 0.78). Out of 120 patients, overall successful graft uptake was seen in 109 (90.8%). In tympanoplasty with microscope, graft take was 90.0%, compared to 91.6% in endoscope group. There was no significant difference in graft take in the two groups.

**Conclusions:**

The tympanoplasty with endoscope is comparable to tympanoplasty with microscope in terms of graft uptake and hearing improvement.

## Introduction

Chronic otitis media (COM) is a common otolaryngologic problem with a significant economic and social burden. In 1878, Berthold did the first tympanic membrane reconstruction; however, Wullstein and Zolhner laid the foundation for tympanoplasty in 1950 (1,2). Tympanoplasty aims to repair the perforated tympanic membrane to render the ear safe from recurrent infections and improve hearing (3). Microscopes have been used traditionally for access to the middle ear and its surgical procedures (4,5). 

However, using a microscope, the problem remains the visualization and approach to middle ear cleft and attic areas for which different approaches have been devised. The introduction of the endoscope revolutionized the field of surgery. In 1992 Guindy et al. published their first article on successful tympanoplasty with endoscope (6). 

The introduction of the endoscope in ear surgery made the middle ear access easy and helped understand the middle ear’s ventilation pathways. It does not mean that endoscope is replacing the microscope, but surgeons using endoscopes advocate using an endoscope for the middle ear and microscope to clear the disease from the mastoid. In addition, better visualization and optics, a broad view with wide-angled endoscopes, and the availability of angle scopes make this instrument more suitable for the middle ear (7).

Limited data compares the success rates and hearing improvement between tympanoplasty with endoscope and tympanoplasty with microscope, particularly in the developing countries where the burden of COM is high. Many alternative surgical approaches, different grafting techniques, and graft materials have also been introduced with time. Temporalis fascia is still the most common graft material used because of its easy access, accessibility, and high success rate (8,9).

This study compares results of tympanoplasty done with microscope vs. endoscope in terms of graft take rate and improvement in conductive hearing loss. 

## Materials and Methods

We reviewed records of 133 patients who underwent tympanoplasty with endoscope and tympanoplasty with microscope type I at Liaquat National Hospital from January to December 2020. We excluded thirteen patients due to incomplete data. We included sixty patients in the microscopic group and sixty in the endoscopic group. Before including patients in the study, informed written consent was taken from all the patients when they came for follow-up. We excluded patients having cholesteatoma, mixed hearing loss on PTA, previous history of ear surgeries, patients requiring ossicular chain reconstruction, and those with missing data. Membrane perforation size was classified objectively on examination if less than 25% tympanic membrane was perforated, it was considered as small, medium (25–50% perforation area), large (50-75% perforation area), and >75% of tympanic membrane involvement was considered subtotal/total. 

Amoxicillin plus clavulanic acid was used in both groups as a prophylactic antibiotic. The first dose was given intravenously before the surgery with an oral five-day course postoperatively.All sixty cases were performed with a conventional postaural approach in the microscope group. 

Temporalis fascia was harvested for graft in all patients. In all patients ossicular chain was examined for discontinuity. Bismuth Iodoform Paraffin Paste (BIPP) pack was placed in the external auditory canal for two weeks. In all patients, postaural sutures were removed on the seventh postoperative day. In the endoscope group, tympanoplasty was done with a permeatal approach with a 4 mm, 18 cm endoscope assisted with a camera system. 0-degree and 45-degree scopes were used. The ossicular chain was examined for continuity in all cases. 

Temporalis fascia for graft was harvested with a small postaural incision and closed in 2 layers. BIPP pack was placed in the external auditory canal for two weeks. Postaural sutures were removed on the seven^th^ postoperative day. 

Data regarding age, gender, laterality, size of perforation, preoperative PTA, the technique of tympanoplasty, postoperative outcomes including graft uptake, and changes in PTA were recorded. Data were entered and analyzed in SPSS version 23.0. Improvement in the air-bone gap was calculated by subtracting the postoperative air-bone gap on PTA from the preoperative air-bone gap for each patient individually to constitute a variable air-bone gap closure. T-test was used to compare the mean pre and postoperative ABG collectively and in endoscopic and microscopic techniques separately and to compare the air-bone gap improvement between the two groups. 

A Chi-square test was used to analyze tympanoplasty techniques and the graft site. A P-value of ≤0.05 was considered significant where applicable. 

## Results

A total of 120 patients were included in the study, and their mean age was 32.43 years (SD ± 10.63) with a range of 15 to 57 years. 66 (55%) patients were female compared to 54 (45%) males. Characteristic features of perforation are given in Table 1. No statistically significant correlation was found between perforation size and graft take rate (P-value 0.07).

**Table 1:** Characteristic features of perforation

**Characteristic feature**	**Frequency (n)**	**Percentage (%)**
Site		
Right	73	60.8%
Left	47	39.2%
***Size***		
Small central	24	20%
Medium central	41	34.2%
Large central	30	25%
Subtotal / Total	25	20.8%

The microscope group was compared with the endoscope group for non-test variables: age, preoperative air-bone gap, and perforation size to see if both groups are comparable. There was no significant difference found between the two groups for non-test variables.

Overall mean preoperative hearing loss was 30.24 (±9.61) dB and mean postoperative hearing loss was 19.36 (±8.54) dB, and the difference was significant (P-value <0.001). When we compared the patients undergoing Type I tympanoplasty by endoscope, the preoperative hearing loss was 29 (±10.41) dB, and the mean postoperative hearing loss was 18.02 (±8.23) dB. In the microscope group, mean preoperative hearing loss was 31.48 (± 8.65) dB, and mean postoperative hearing loss was 20.70 (± 8.7) dB. Mean air-bone gap closure was 10.98 (± 4.62) dB for the endoscope group while 10.98 (±4.21) dB for the microscope group. There was no statistically significant difference in air-bone gap closure between the two groups (P-value 0.78) (Table 2).

**Table 2:** Comparison of hearing loss

	**Tympanoplasty with endoscope**	**Tympanoplasty with microscope**
Mean pre-operative hearing loss	29 (±10.41) dB	31.48 (± 8.65) dB
Mean postoperative hearing loss	18.02 (±8.23) dB	20.70 (± 8.7) dB
Mean air-bone gap closure	10.98 (± 4.62) dB	10.98 (±4.21) dB

Out of 120 patients, overall successful graft uptake was seen in 109(90.8%) patients, and it was not significantly associated with the technique of surgery (endoscopic vs. microscopic) (Table 3).

**Table 3:** Postoperative graft uptake

	**Postoperative tympanic membrane**		
	Intact	Perforation	Difference (P-value)
*Technique*			1.00
Endoscopic	55(91.6%)	5(8.3%)	
Microscopic	54 (90.0%)	6 (10.0%)	

## Discussion

Tympanic membrane perforations were successfully repaired in 90.0% of tympanoplasty with microscope and 91.6 % of tympanoplasties with endoscope. Similar success rates have been reported previously, ranging from 83.3-100% and 82.4-100%, respectively (10-12). 

Tseng et al. conducted a meta-analysis and said that graft success rates were 86.4% and 85.1%, without a significant difference between tympanoplasty with microscope and tympanoplasty with endoscope (13).

The perforation size did not affect the graft success rate and post-op air-bone gap closure. Tseng et al. and Ayache et al. reported that the perforation size does not affect graft uptake and hearing restoration (14,15). Our study found that air-bone gap closure was statistically significant in both groups after surgery (P<0.001). We also found no statistically significant difference between the endoscope and microscope groups in hearing improvement. The result of our study is comparable to previous studies by Dunder et al., Huang et al., Sinha et al., who also found that postoperative ABG improvement is statistically significant after tympanoplasty regardless of which technique was used (16-18). In addition, there was no significant difference in air-bone closure for microscopic technique and endoscopic technique.

In this study, patients who underwent Type I tympanoplasty were selected to make the two groups comparable and control the confounders. Otherwise, endoscopes are more valuable to address patients with attic retraction pockets and limited cholesteatoma with a permeatal approach. This study provides added evidence to accept the endoscope as another helpful tool for ear surgery. In this study, 4.0mm and 18cm endoscopes were used, similar to those routinely used in endoscopic nasal surgeries. Thus, adopting this new technique is swift and easy for surgeons doing endoscopic nasal procedures routinely, With its short-term follow-up, our data fills the gap in literature from developing countries where otitis media is more prevalent. However, there are a few limitations. Firstly, we followed the patients prospectively but reviewed surgical notes in retrospect, so data analysis did not reveal exact pathology. Nevertheless, we assume that patients who had central perforation and underwent simple Type I tympanoplasty had similar pathology. However, both groups had no statistical difference in the type of perforation and the preoperative air-bone gap that helped reduce the baseline difference in pathology characteristics.

## Conclusion

In conclusion, tympanoplasty with endoscope is comparable to tympanoplasty with microscope in terms of graft success and hearing outcomes for patients needing type I tympanoplasty. Further studies are needed to evaluate endoscopes for other types of tympanoplasty and ossiculoplasty. 
